# Long-Term Monitoring of a Tunnel in a Landslide Prone Area by Brillouin-Based Distributed Optical Fiber Sensors †

**DOI:** 10.3390/s21217032

**Published:** 2021-10-23

**Authors:** Aldo Minardo, Ester Catalano, Agnese Coscetta, Giovanni Zeni, Caterina Di Maio, Roberto Vassallo, Luciano Picarelli, Roberto Coviello, Giuseppe Macchia, Luigi Zeni

**Affiliations:** 1 Department of Engineering, University of Campania “Luigi Vanvitelli”, 81031 Aversa, Italy; ester.catalano@unicampania.it (E.C.); agnese.coscetta@unicampania.it (A.C.); luciano.picarelli@unicampania.it (L.P.); luigi.zeni@unicampania.it (L.Z.); 2 Institute for Electromagnetic Sensing of the Environment, Italian National Research Council, 80124 Naples, Italy; zeni.g@irea.cnr.it; 3 School of Engineering, University of Basilicata, 85100 Potenza, Italy; caterina.dimaio@unibas.it (C.D.M.); roberto.vassallo@unibas.it (R.V.); 4 Rete Ferroviaria Italiana (Ferrovie dello Stato Italiane Group), 70122 Bari, Italy; r.coviello@rfi.it (R.C.); g.macchia@rfi.it (G.M.)

**Keywords:** optical fibers, distributed sensing, remote monitoring, early warning systems

## Abstract

This paper shows the results of the monitoring of the deformations of a tunnel, carried out using a distributed optical fiber strain sensor based on stimulated Brillouin scattering. The artificial tunnel of the national railway crosses the accumulation zone of an active landslide, the Varco d’Izzo earthflow, in the southern Italian Apennines. Severely damaged by the landslide movements, the tunnel was demolished and rebuilt in 1992 as a reinforced concrete box flanked by two deep sheet pile walls. In order to detect the onset of potentially dangerous strains of the tunnel structure and follow their time trend, the internal deformations of the tunnel are also monitored by a distributed fiber-optic strain sensor since 2016. The results of the monitoring activity show that the deformation profiles are characterized by strain peaks in correspondence of the structural joints. Furthermore, the elongation of the fiber strands crossing the joints is consistent with the data derived by other measurement systems. Experiments revealed an increase in the time rate of the fiber deformation in the first and last part of the monitoring period when the inclinometers of the area also recorded an acceleration in the landslide movements.

## 1. Introduction

In the last decades, there has been a growing interest in the use of distributed optical fiber sensors in the field of structural and geotechnical engineering. These sensors offer the unique advantage of detecting the strain distribution over an extended area and at high spatial resolution [1,2,3]. Furthermore, they share the advantages of optical fiber sensors, such as small size, light weight, corrosion resistance, ease of encapsulation and implementation. Distributed optical fiber sensors are usually based on some form of scattering of the light propagating in the fiber, specifically Raman scattering, Rayleigh scattering, and Brillouin scattering. Distributed optical fiber sensors based on Brillouin scattering feature extremely long sensing distances (up to several tens of km); therefore, they are typically preferred for the monitoring of large structures from remote locations [4,5,6].

In recent years, several experiments have demonstrated the reliability and feasibility of Brillouin sensing technology for structural health monitoring. Gao et al. [7] measured the strain of post-tensioning aramid fiber reinforced plastic (AFRP) cables, using a specialty optical fiber sensor bonded to a steel strand. The monitoring was performed both in the tension procedure and load procedure. It was found that the discrepancy of the strains measured by the optical fiber sensor and by a conventional strain gauge was very limited. Shi et al. [8] applied the same sensing technology to monitor the deformation of a tunnel in the field, using FRP materials to reinforce the optical fiber sensor. Hisham et al. [9] employed the Brillouin scattering technology to monitor the strain and temperature along a bored pile adjacent to a deep excavation. A simplified calculation method was proposed for converting the measured strain into displacements. The monitoring results demonstrate that the distributed sensor can supply reliable data for calculating the deflection of bored piles.

Piles, diaphragm walls, and tunnel linings are all important components of underground structures. During the construction of piles, load tests are important for investigating their actual field behavior. The continuous measurement of deformation along the pile, offered by distributed optical fiber sensors, helps to understand the real pile-soil interaction behavior [10]. Furthermore, thanks to their capability to detect both compressive and tensile strain with high sensitivity, distributed optical fiber sensors can be useful in monitoring early movements of soil slopes by the direct embedding of suitable fiber cables in the ground [11,12]. Laboratory scale tests have been performed, showing the capability of optical cables, properly deployed along the slope, to detect the early ground movements (settling and sliding) caused by an artificially induced rain, well before the onset of the landslide [13,14,15,16,17,18].

The optical fiber technology has also been applied for the realization of fiber-optic inclinometers, where the fiber is glued along parallel directions of the tube. It has also been shown that, by processing the acquired deformations, a three-dimensional reconstruction of the displacement distribution can be obtained [19,20]. Compared to conventional inclinometers, fiber-optic inclinometers provide a continuous deformation profile and measure a larger deformation of the tube before getting out of use.

In this work, we present the results of a monitoring campaign carried out in a railway tunnel using a Brillouin based distributed optical fiber sensor. The measurement method is the same as described in [21,22]. In detail, the sensor head is a conventional and low-cost optical fiber, with a tight buffer of 0.9 mm, simply glued along the sidewalls of the tunnel. The tunnel is in an area in the South of Italy where an active landslide moves at an average rate of 1 cm/year [23]. The original structure was severely damaged by the landslide movements. In 1992, the tunnel was rebuilt and, since then, carefully monitored by inclinometers installed in the subsoil, upslope and downslope from the tunnel. The new tunnel, whose internal geometry was reconstructed by 3D laser scanning [23], has a reinforced concrete box structure with the bottom slab, roof, and vertical walls cast in place such that they have an effective connection among themselves, providing increased stiffness and strength against landslide movements. In 2016, the optical fiber system was installed along the tunnel sidewalls in order to monitor the local displacements induced by the landslide. We note that, while distributed optical fiber sensing technology has been well known for several years, relatively few reports have been published about the use of this technology for the long-term monitoring of large structures such as the railway tunnel considered in this work [8,24,25].

In the following, after briefly recalling the principles of optical fiber distributed sensing based on stimulated Brillouin scattering (SBS), the results of the monitoring campaign will be presented and discussed. The results indicate that the optical fiber sensor can detect localized cracks, identify their location along the tunnel walls, and follow their evolution over time. Furthermore, the comparison between the displacements derived from the optical fiber strain measurements and the displacements provided by inclinometers installed in the proximity of the tunnel, and satellite SAR data, demonstrate the congruence between the data provided by the optical fiber sensor and those from conventional monitoring systems.

## 2. Brillouin Optical Time Domain Analysis for Distributed Sensing

The technology employed in our tests to measure the strain distribution along the tunnel sidewalls relies on the stimulated Brillouin scattering (SBS) phenomenon. In single-mode optical fibers, SBS is observed by injecting two frequency-shifted beams at the two opposite ends [26]. The superposition between the two beams gives rise to an interference pattern, moving along the fiber at a velocity proportional to the frequency shift between the two beams. When the frequency shift falls within the so-called Brillouin Gain Spectrum (BGS) of the fiber, an intense acoustic wave is created by electrostriction. The acoustic wave acts as a diffraction grating, scattering part of the energy of the pump beam (the one having the highest frequency) in the backward direction. The scattered light adds energy to the probe wave (the one having the lowest frequency), which, therefore, becomes amplified as it interacts with the acoustic wave. The probe gain is governed by the BGS, being maximum at the so-called Brillouin Frequency Shift (BFS), which is ≈10–11 GHz in single-mode optical fibers at the wavelength λ = 1.55 µm. As the BFS is linearly dependent on strain and temperature, the latter two can be determined from it. In the so-called Brillouin Optical Time-Domain Analysis (BOTDA), one of the two optical beams (the pump wave, typically) is pulsed, thus that the SBS interaction occurs sequentially, in different portions of the fibers, thus it can be temporally resolved. By recording the intensity of the probe wave emerging from the sensing fiber as a function of time, the spatial distribution of the Brillouin gain can be determined. Furthermore, repeating the measurements for several pump-probe frequency shifts, the Brillouin gain spectrum (BGS) can be determined at each fiber position with sufficient spectral granularity. The spatial resolution is proportional to the pump pulse duration, with a 10 ns pulse duration giving rise to a 1 m spatial resolution.

**Figure 1:**
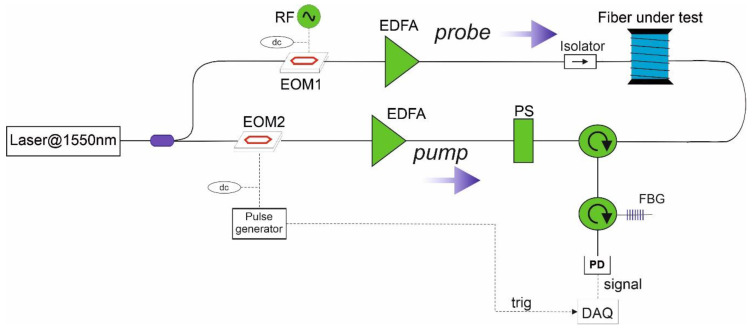
Experimental set-up for (BOTDA) Brillouin Optical Time-Domain Analysis measurements. (EOM) electro-optic modulator; (PS) polarization switch; (EDFA) erbium-doped fiber amplifier; (PD) photodetector; (FBG) fiber Bragg grating; (DAQ) data acquisition (redrawn from [21]).

The field trials presented in this work have been carried out using a prototypal BOTDA analyzer implementing the scheme in Figure 1 [21].

In this scheme, both pump and probe waves were generated from the same external cavity laser source (CoBrite DX1 from ID Photonics, Neubiber Germany), featuring a linewidth of 100 kHz and emitting a power of 40 mW at the wavelength of 1.55 µm. The laser light was first split in two branches by means of a 50/50 optical coupler. In the upper branch, the light was frequency-shifted through an electro-optic modulator (EOM1) driven by a RF signal close to the BFS of the fiber. The DC bias of the electro-optic modulator was set to the minimum of its transfer function, therefore, realizing a dual sideband, suppressed-carrier modulation. The light exiting from EOM1 has two spectral lines, one upshifted and the other one downshifted from the laser frequency by a quantity equal to the RF modulation frequency. The so-formed probe light was launched into one end of the fiber under test (FUT) after being passed through an optical isolator. When interacting with the pump light, the lower sideband becomes amplified from the SBS interaction, whereas the upper sideband becomes depleted [27]. In the lower branch, the pump light was pulsed by another electro-optic modulator (EOM2), driven by an electrical pulse generator, and biased at the minimum transmission. The scheme also includes a polarization switch (PS), allowing the measurement of the Brillouin gain for two orthogonal state-of-polarizations (SOP) of the pump light. In fact, summing together the two measurements, one gets rid of the Brillouin gain dependence on the SOP of the interacting signals. The probe light transmitted over the fiber is sent to a narrowband (≈4 GHz) fiber Bragg grating (FBG), reflecting only the lower sideband of the probe light (i.e., the Stokes line). The FBG also filters out the amplified spontaneous emission (ASE) of the erbium-doped fiber amplifier (EDFA) inserted into the probe branch. The probe light emerging from the FUT is detected through a photodetector with a bandwidth of 125 MHz and a conversion gain of 4 × 10^4^ V/W. Data acquisition is carried out by a fast analog-to-digital converter connected to a dedicated Field Programmable Gate Array (FPGA) processor for real-time averaging. The acquisition is performed by scanning the RF applied to EOM1 to a proper range, thus allowing the BGS to be reconstructed at each fiber position. Finally, the acquired data are post-processed by applying a cross-correlation method to each acquired BGS [28], providing an estimate of the BFS in each position.

For the experiments reported in the following, the BOTDA measurements were carried out at a spatial resolution of 1m, with an estimated strain resolution of ±10 µε.

## 3. The Landslide

**Figure 2:**
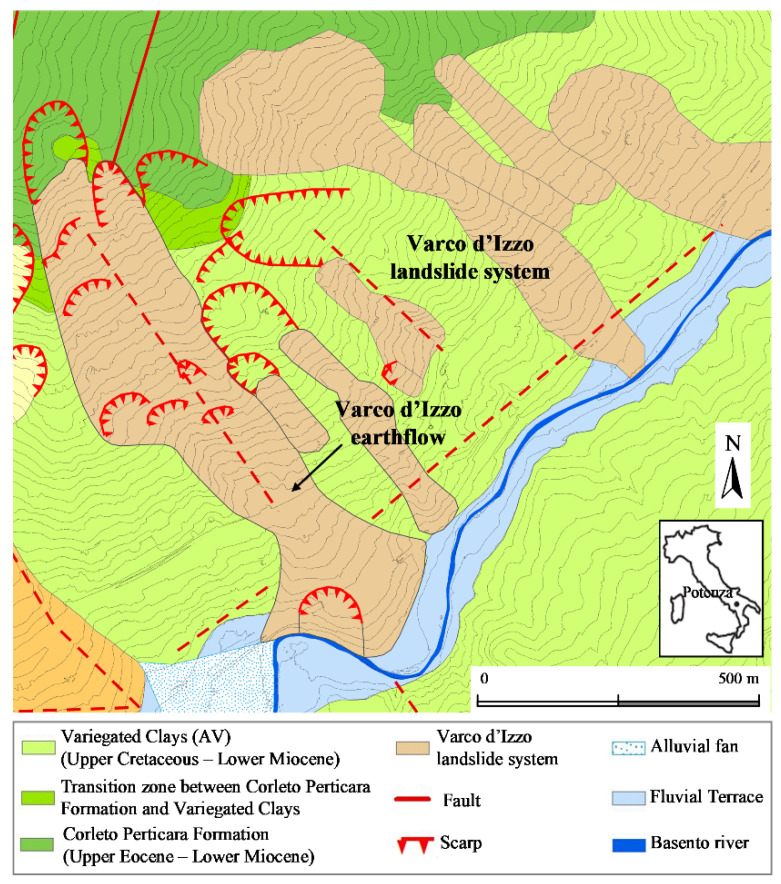
Geological map of the Varco d’Izzo landslide system (redrawn from [29]).

The landslide system crossed by the tunnel is located in the eastern suburbs of the town of Potenza (Southern Italy) (Figure 2). It develops within the Variegated Clay Formation (Upper Cretaceous–Lower Miocene), constituted by a succession of chaotic, heterogeneous, severely tectonized scaly clays and marly clays. The main earthflow develops along a NW-SE fault in the most urbanized zone of the slope. The fault has probably been the cause of the first slope failure and subsequent landslide evolution. The landslide is characterized by an average inclination of about 10°, approximately 1250 m in length, 150 to 300 m in width, and multiple slip surfaces [29]. In the accumulation zone, a local slide occurs in correspondence to a bend of the Basento river. The earthflow body is constituted by a mixture of different lithotypes: rock fragments, blocks, and disarranged strata of marly limestone and calcarenite are incorporated in a clayey matrix, which governs the landslide behavior. The clay fraction (c.f.) ranges between 25% and 50%, the liquid limit w_L_ between 50% and 100%, while the residual friction angle ranges between 6° and 13°. The tunnel crosses the accumulation zone, close to the Basento river, which is subjected to localized erosion.

## 4. Monitoring Results

### 4.1. Landslide Displacements

To evaluate the time evolution of the displacements of the area under study, different experimental devices were used in the last 30 years. Deep and superficial displacements were monitored by several institutions, mainly by mobile probes inserted in inclinometer tubes.

**Figure 3:**
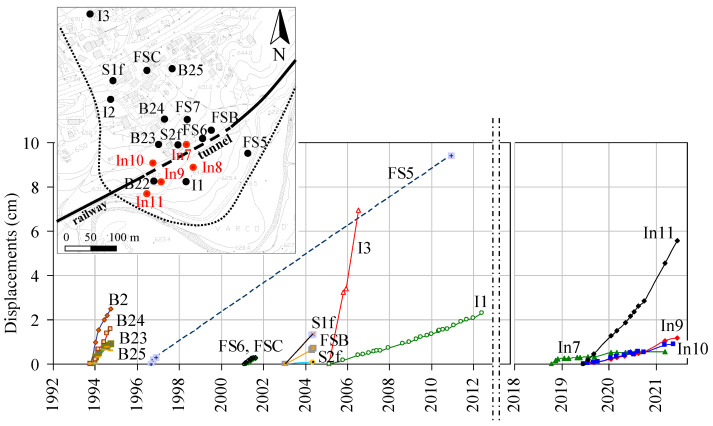
Deep displacements against time obtained from inclinometer data of Varco d’Izzo earthflow. Letters B, S, FS, I indicate inclinometers installed in different monitoring campaigns since 1992.

In the area, data from 19 inclinometers installed from 1993 to 2006 were available, reaching a maximum depth of 50 m. However, now all these inclinometers are out of use because of excessive cumulated shearing. The most recently installed set of sensors, still in use, consists of 5 inclinometer tubes, with horizontal displacement measurements generally carried out on a monthly basis. Readings along the height of the tubes were taken in steps of 0.5 m (equal to the distance between the two spring-pressured wheels of the servo-accelerometer probe), and their precision was 1 mm per 20 m. Figure 3 reports all displacement data against time. The average annual rates were highly variable from one zone to another while being in the order of one to a few cm/year. Displacement rates of the more recently installed inclinometers were similar to those of inclinometers installed in the early 2000s. Therefore, from the whole displacement dataset, no significant acceleration was observed in the last 20 years. In particular, inclinometer I1, located in the landslide accumulation, presents the longest and more detailed data series, and shows an annual rate fundamentally constant from 2005 to 2012.

Fixed GPS stations with continuous acquisition and benchmarks for periodic measurements were installed in 2006 and have provided displacement data since then. Details about installation, data acquisition, and displacement time series registered from 2006 to 2020 are reported in [29]. SAR COSMO-SkyMed (CSK) satellite images of years 2011–2014 were also processed and interpreted [29].

CSK satellite SAR data relative to the period 2011–2014 were processed by differential interferometry techniques within the PST-A (in Italian: Piano Straordinario di Telerilevamento Ambientale), i.e., the high precision plan of remote sensing of the Italian Ministry of Environment [30]. As well known, such data provide the component of superficial displacements, detected on permanent scatterers, along the satellite Line-Of-Sight (LOS) [31,32,33]. By comparison with ground-based displacement data, after careful assessment of total displacement azimuth and inclination to the horizontal, the component along the LOS can be converted to horizontal displacements and, therefore, compared to inclinometer or GPS data [29,34].

**Figure 4:**
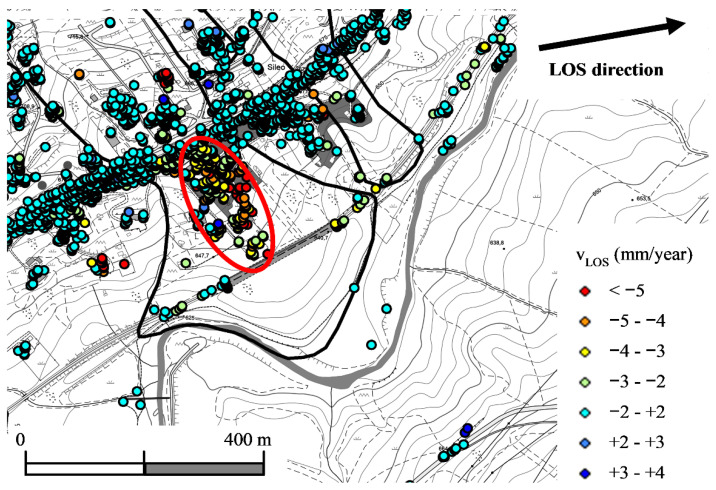
Average displacement rates in the period May 2011–March 2014 evaluated from COSMO-SkyMed ascending data for the accumulation of Varco d’Izzo earthflow (redrawn from [29]).

**Figure 5:**
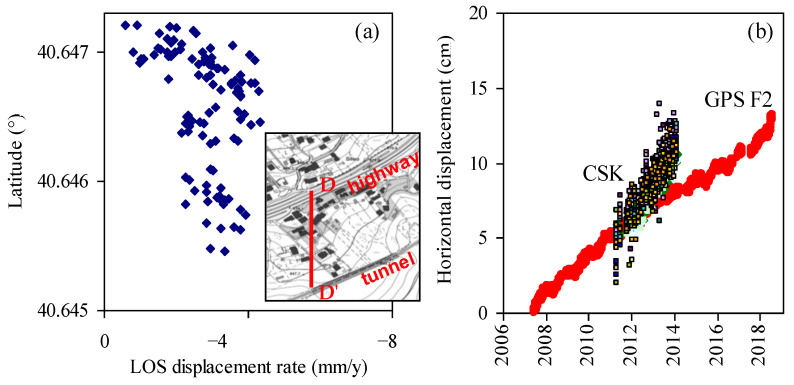
(**a**) Average Line-Of-Sight (LOS) displacement rates in the period May 2011–March 2014 along section DD’; (**b**) comparison with GPS data obtained in the same area (redrawn from [29]).

In the zone between the highway and the railway tunnel, displacement data were rather uniform, with a rate along the LOS of about 3 mm/year, as shown by the map in Figure 4 and the distribution along section DD’ in Figure 5a. Horizontal displacement rates were approximately three times the LOS component. The rates calculated from CSK satellite data were similar to (and, in particular, slightly higher than) those of fixed GPS station F2 with continuous acquisition, as shown by Figure 5b.

### 4.2. Optical Fiber Monitoring

**Figure 6:**
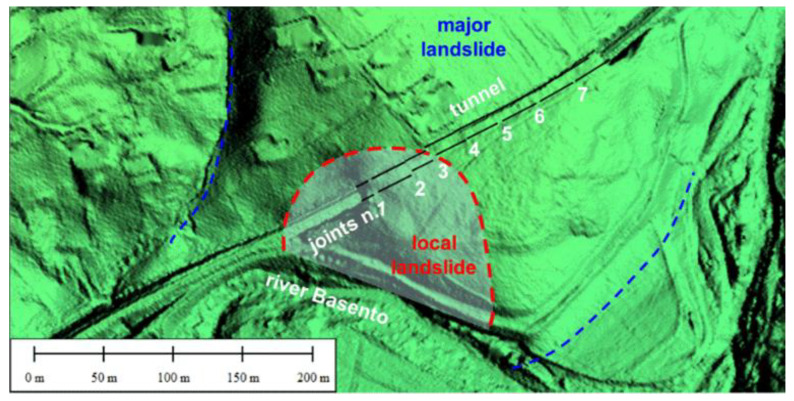
Plan view of the landslide accumulation and of the monitored tunnel (redrawn from [21]).

The BOTDA sensor presented in Section 2 was employed to detect the strain distribution along the two sidewalls of a 200 m long railway tunnel (tunnel “Calabrese”), consisting of eight contiguous sectors separated by joints with irregular spacing (the average length of each sector being 25 m). Figure 6 shows the tunnel location in the landslide area and the position of the construction joints. 

**Figure 7:**
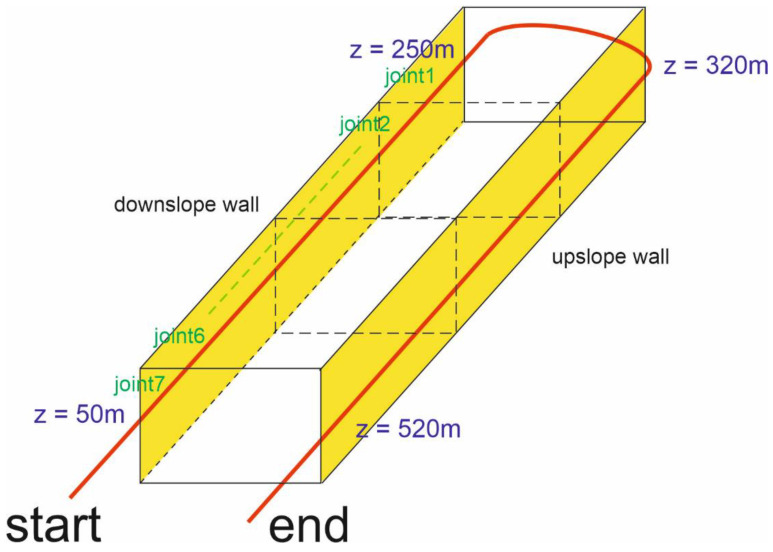
Path (shown in red) of the optical fiber installed along the tunnel sidewalls.

As the sensing element, a single-mode 0.9 mm tight-buffered optical fiber was hand-glued along the two sidewalls of the tunnel using epoxy adhesive. The optical fiber was a G657 low bending loss fiber, with a BFS of ≈10.7 GHz at room temperature. As the adopted measurement scheme requires access from both ends of the sensing fiber (see Figure 1), the two strands of fiber running along the two sidewalls were spliced together at one of two entrances of the tunnel, realizing a single, uninterrupted optical path (see Figure 7). In Figure 7, START indicates the launching section for the pump wave, while END indicates the launching section for the probe. The figure also reports the approximate abscissas of the sensing fiber at the beginning and the end of the two sidewalls.

**Figure 8:**
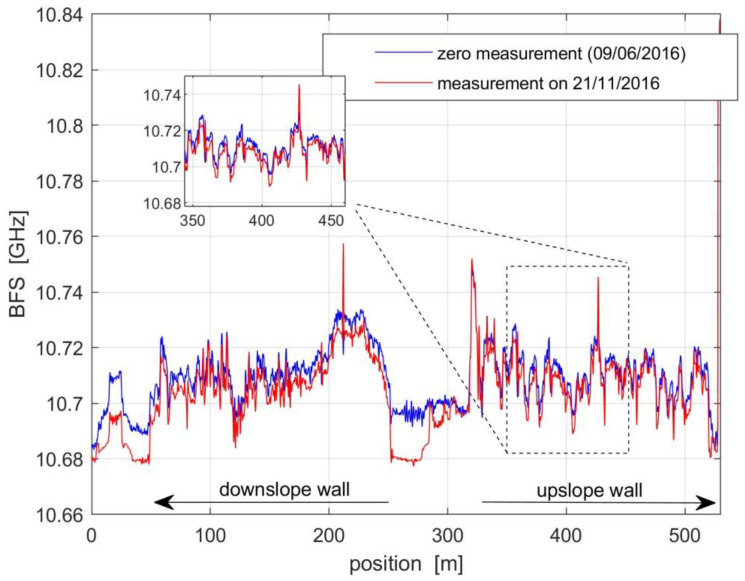
BFS measurements taken on 6 June 2016 (blue line) and 21 November 2016 (red line). The inset shows a zoomed view of the acquired BFS profiles from ≈350 m to ≈450 m.

A reference acquisition of the BFS profile along the whole fiber was carried out on 9 June 2016. This measurement was then stored and subtracted from the successive records. The BFS changes were then converted into strain, adopting a standard transduction factor of 20 με/MHz. As an example, we compare in Figure 8 the reference data, as well as the data taken on 21 November 2016.

We see that the BFS profiles were spatially irregular. This should be attributed to the strain induced on the optical fiber by the gluing operation. However, the inset of Figure 8 reveals that these irregularities remained constant over time. Therefore, they can be easily compensated by subtraction with the reference profile. We also note that the central part of the profiles corresponds to the portion of the fiber employed to connect the downslope and upslope fibers. In that region, the optical fiber ran outside the tunnel and, therefore, was more affected by the ambient temperature. The temperature inside the tunnel was more stable, even though some seasonal temperature variation was observed as well. In order to isolate the effects of the temperature from the strain in the acquired BFS profiles, an offset was applied to each BFS profile in order to minimize the root mean square (r.m.s.) difference between consecutive BFS measurements over each tunnel sidewall. This offset was separately calculated for each BFS measurement. This method assumes that the temperature was uniform inside the tunnel, which has been verified as a reasonable approximation.

**Figure 9:**
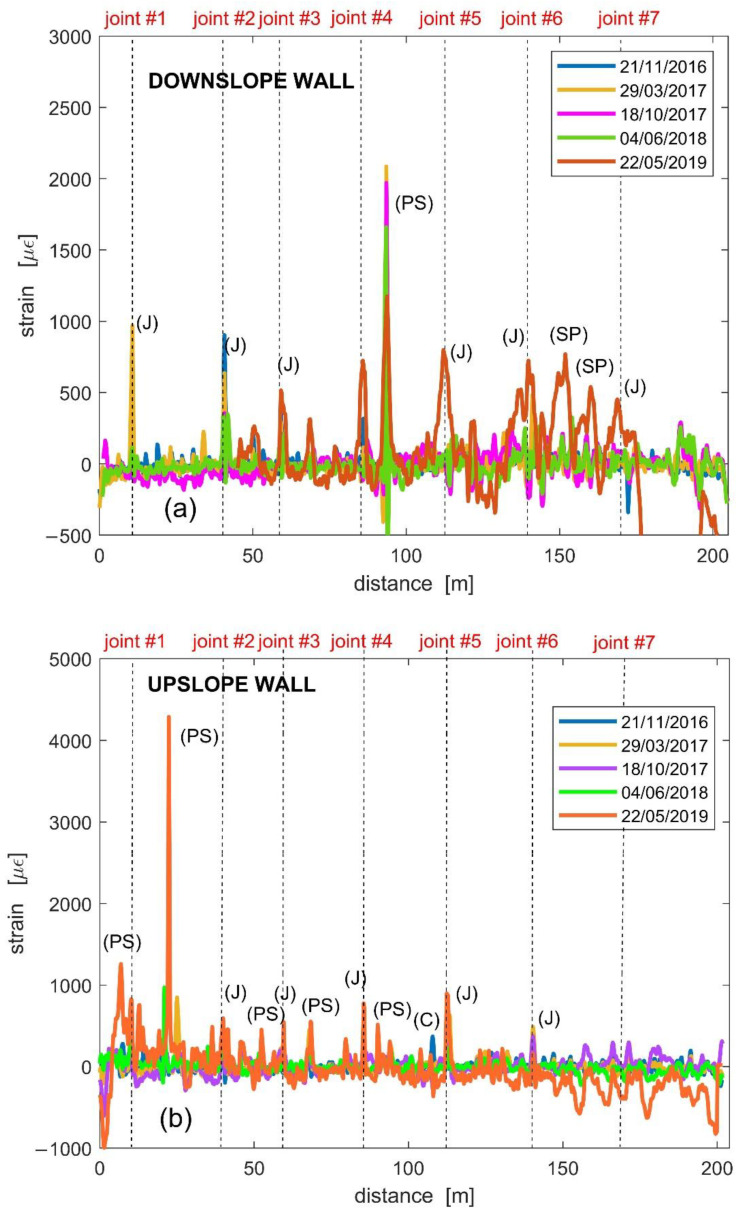
Results of the optical fiber strain measurement along the (**a**) downslope and (**b**) upslope tunnel wall, with indication of the position of joints. J—Joint, PS—local Parget Swelling, SP—Salt Precipitate accumulation, C—Crack.

Figure 9a,b report the results relative to the whole monitoring campaign, separately shown for the upslope and downslope tunnel sidewalls, respectively. Note that, the strain distribution along the downslope wall has been flipped (compared to the optical fiber raw measurement), to show the variations along the same direction as the upslope profile (i.e., from joint #1 to joint #7 in Figure 6 and Figure 7). The vertical dashed lines in the same figure indicate the position of the structural joints. We also observe that, in the last measurement of 22 May 2019, the first 45 m of the downslope profile were missing due to breakage of the fiber in that region. Note that the measurement taken on 22 May 2019 was in addition to the data previously reported in [21].

**Figure 10:**
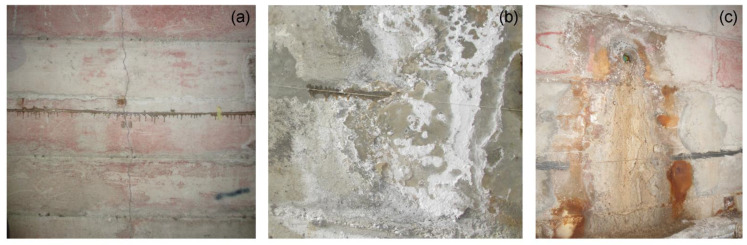
Crack on the tunnel wall crossed by the glued fiber (**a**); parget swelling and salt precipitate accumulation (**b**); salt precipitate accumulation (**c**).

The strain profiles exhibit several peaks, which, is some cases, occur in the position of the structural joints. Through careful inspection of the tunnel conditions, we can explain the origin of these peaks in terms of four different phenomena, indicated by a bracketed letter in Figure 9a,b. The letters C, PS, and SP correspond, respectively, to a crack, a local parget swelling on the tunnel walls, and a salt precipitate accumulation due to water infiltration and evaporation, exemplified in Figure 10. The letter J corresponds to the joints where relative displacements of the tunnel sectors occur. 

Figure 9a,b show that the highest strain was caused by the local parget swelling. However, the most interesting results were related to the joints’ opening/misalignment deformation peaks. The highest fiber strains mostly occurred in correspondence of joints 1, 2, and 3, where the most appreciable relative displacements have been measured since the tunnel construction in 1992 until recent years by caliper. In particular, approximately 2 cm misalignment was observed in joint n. 3 and slightly lower values in joints n. 1 and 2 [21].

**Figure 11:**
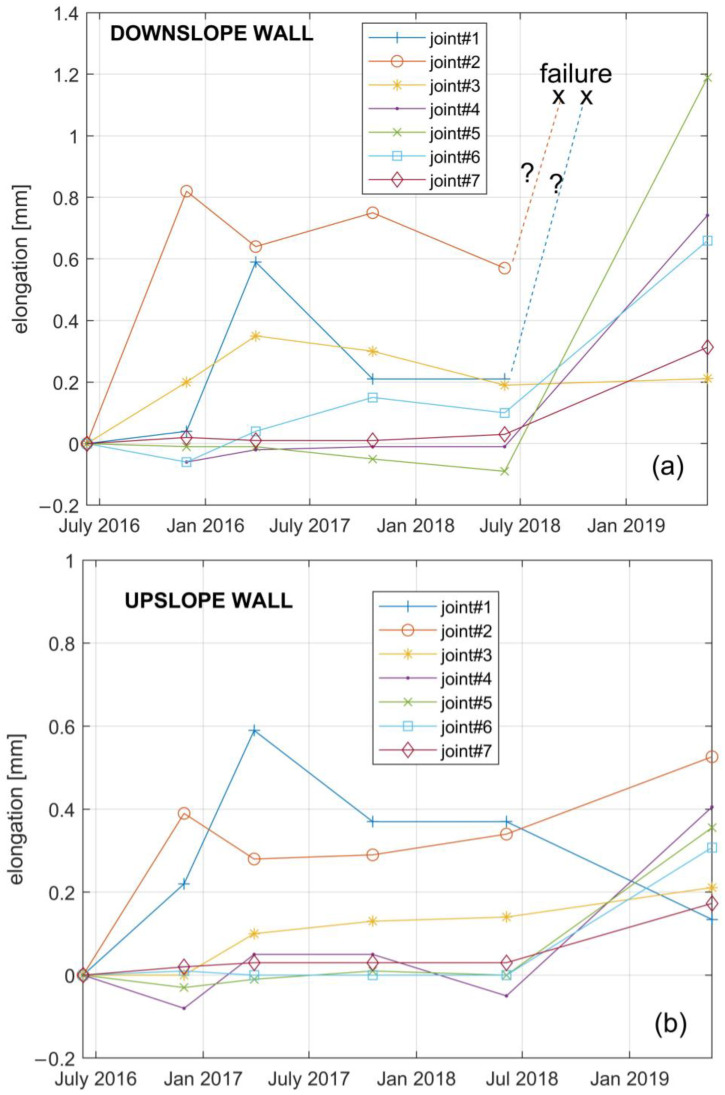
Elongation against time of the optical fiber, corresponding to the tunnel joints along the (**a**) downslope or (**b**) upslope sidewall.

In order to estimate the resulting fiber elongation corresponding to the joint’s opening, we followed the procedure described in [21]. In brief, the strain values around each J peak (Figure 9) were detrended and integrated along a fiber length corresponding to the spatial resolution of 1 m. Assuming a strain resolution of ±10µε and a sampling step of 40 cm along the fiber, the corresponding elongation resolution over the spatial resolution was ±17 µm. Figure 11a,b show the time series of such fiber elongation in both the upslope and downslope tunnel walls.

## 5. Discussion

Figure 9 shows that the strain peak values along the downslope wall are higher than those along the upslope wall. A similar result is provided in terms of fiber elongations by Figure 11. This is consistent with the local kinematics of the landslide accumulation that opens like a fan towards the river, which, in turn, triggers the movements by erosion [29]. The data of Figure 11 show that in the first phase of the measurements, i.e., the second half of 2016, the fiber underwent elongations in correspondence of joints 1, 2, and 3, which in fact fall into the area where the small local landslide triggered by river erosion develops (Figure 6). After the measurement of June 2018, at about two years from the installation, the fiber underwent elongation in all the joints, and it underwent even failure in the downslope joints 1 and 2. In order to perform the subsequent measurement in the rest of the fiber in May 2019, it was necessary to realize a bypass of these joints. In other words, a piece of fiber was spliced between the fiber attached along the downslope wall after joint 3, and the fiber attached along the upslope wall before joint 1. Note that, adopting a Brillouin Optical Time-Domain Reflectometry (BOTDR) scheme, the system could still be operating even after a fiber breakage, as the measurement requires access to only one end of the optical fiber [35].

The global time trend of fiber elongations appears in agreement with that of the landslide displacements estimated by GPS measurements. In fact, the data of station F2 in Figure 5 and further data reported by Vassallo et al. [29] showed lower displacement rates in the period June 2016–June 2017, consistently with a decrease in the local cumulated annual rainfall. Furthermore, a recent analysis of Cosmo-SkyMed data [34] showed an acceleration of the movements in the accumulation area between 2018 and 2020, when fiber failure occurred in correspondence of joints 1 and 2. The experimental results thus demonstrate the reliability of the fiber system to monitor the strain distribution and the tunnel behavior.

## 6. Conclusions

We have reported the results of a three-year monitoring campaign of a railway tunnel crossing the accumulation zone of an active landslide in Southern Italy. The zone is being monitored by different technologies (GPS, satellite, inclinometers, 3D laser scanning), including distributed optical fiber sensors—glued to the tunnel walls—based on stimulated Brillouin scattering. The data show that the landslide accumulation is moving extremely slowly upslope and downslope from the tunnel. The same tunnel is apparently able to contrast the earth thrust with limited deformations. The optical fiber technology has proven able to successfully monitor such deformations with high resolution. Given the possibility of spatial and temporal continuous measurements and remote operation, the monitoring system has thus the potential of detecting and pinpointing structural anomalies occurring to the tunnel, acting as a reliable early warning system. 

## Data Availability

The BOTDA datasets analyzed during the current study are available from the corresponding author on reasonable request.
